# The Emerging Role of Adiponectin in Female Malignancies

**DOI:** 10.3390/ijms20092127

**Published:** 2019-04-30

**Authors:** Luca Gelsomino, Giuseppina Daniela Naimo, Stefania Catalano, Loredana Mauro, Sebastiano Andò

**Affiliations:** 1 Department of Pharmacy, Health and Nutritional Sciences, University of Calabria, 87036 Arcavacata di Rende (CS), Italy; lugelso@gmail.com (L.G.); giuseppinadanielanaimo@gmail.com (G.D.N.); stefcatalano@libero.it (S.C.); 2 Centro Sanitario, University of Calabria, Via Pietro Bucci, 87036 Arcavacata di Rende (CS), Italy

**Keywords:** obesity, adipokines, adiponectin, breast cancer, ovarian cancer, endometrial cancer, cervix cancer, estrogen receptor

## Abstract

Obesity, characterized by excess body weight, is now accepted as a hazardous health condition and an oncogenic factor. In different epidemiological studies obesity has been described as a risk factor in several malignancies. Some biological mechanisms that orchestrate obesity–cancer interaction have been discovered, although others are still not completely understood. The unbalanced secretion of biomolecules, called “adipokines”, released by adipocytes strongly influences obesity-related cancer development. Among these adipokines, adiponectin exerts a critical role. Physiologically adiponectin governs glucose levels and lipid metabolism and is fundamental in the reproductive system. Low adiponectin circulating levels have been found in obese patients, in which its protective effects were lost. In this review, we summarize the epidemiological, in vivo and in vitro data in order to highlight how adiponectin may affect obesity-associated female cancers.

## 1. Introduction

**Figure 1:**
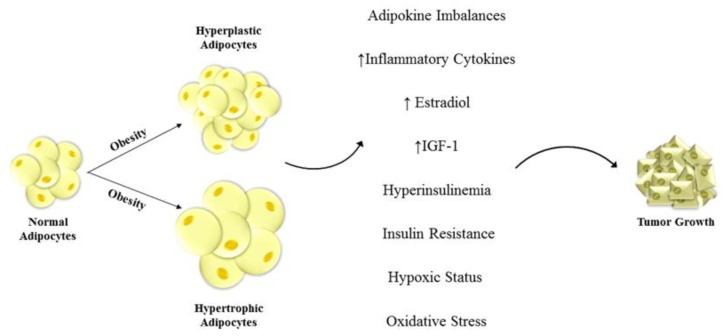
Unraveled mechanisms linking obesity and cancers. Hyperplastic and hypertrophic adipocytes are one of the main features of obesity. This dis-regulation of fat cells leads to change in adipokine and inflammatory cytokine secretion, enhanced insulin-like growth factor-1 (IGF-1) and estradiol production, and to hyperinsulinemia, insulin resistance, hypoxic status and oxidative stress. These alterations in the tumor microenvironment deeply impact the phenotype of the surrounding cells inducing severe modifications in cell behavior that contribute to tumor development and progression.

Worldwide, obesity is spreading and is reaching epidemic proportion, thus becoming a critical public health issue. Today, the World Health Organization (WHO) reported that people with a body mass index (BMI) greater than 30 kg/m^2^ (30.0–34.9, grade I; 35.0–39.9, grade II; and ≥40, grade III) includes almost 1.9 billion adults and this number is rising fast [1]. This pandemic condition is associated with various metabolic disorders, cardiovascular diseases, type 2 diabetes and several cancers [2,3,4,5]. Meta-analyses and several epidemiological studies defined the fitted connection between cancer development and obesity [6,7,8,9,10,11]. In 2011, it has been described that in the United States 85,000 persons per year affected by obesity experienced cancer [10]. Obesity could be considered a risk factor for cervical, ovarian, endometrial and breast cancer, and it has been reported to be responsible for 88% mortality rates in females [7,12]. Insulin resistance and altered insulin-like growth factor-1 (IGF-1) pathway activation, changes in bioavailability of sex hormones and a chronic inflammatory state related to obesity conditions have been recognized to induce cancer development and progression [12,13,14]. Furthermore, obesity alters the secretion of several molecules released by adipocytes, known as adipokines. Among adipose tissue-derived factors, it has been well documented that adiponectin exerts a critical role in the pathogenesis of obesity-associated disorders. It has been reported that adiponectin circulating levels are dramatically decreased in obese patients [15,16] (Figure 1). Indeed, adiponectin expression and secretion is negatively correlated to the BMI, even though the mechanisms responsible for this down-regulation are not yet completely elucidated [17,18,19]. Low circulating levels of adiponectin in overweight women may be related to the blunted chronic inflammatory status in obesity. The enhanced production of tumor necrosis factor α (TNFα) and Interleukin-6 (IL-6), concomitant with the hypoxic status in adipose tissue, represent a possible mechanism involved in the adiponectin down-regulation in obese subjects [20,21]. Many epidemiologic studies established a correlation between hypoadiponectinemia and an enhanced risk of obesity-related disorders [15,22,23,24,25,26].

Nevertheless, there are still hidden molecular mechanisms involved in this relationship that need to be explored. In this review, we will discuss the association of this adipokine with obesity and different female cancers, in which low adiponectin levels confer altered risk and influence progression in affected women. 

## 2. Adiponectin Structure and Biology

Adiponectin is the most abundant adipokine detected in circulating plasma, wherein its concentration ranges from 3 to 30 µg/mL [22,27,28]. Adiponectin is mainly produced and secreted by white adipose tissue and in lower amounts by other tissues such as brown adipose tissue, placenta, fetal tissue, colon, skeletal muscle, salivary glands, and liver [27,28,29,30,31,32,33,34,35,36]. Structurally, adiponectin is a 244 amino acid-long polypeptide with four domains: An N-terminal region, a variable sequence, a collagen-like motif, and a C-terminal globular domain [22,30,37,38]. Adiponectin belongs to the C1q-like protein family, sharing a high sequence homology with the complement factor C1q in the C-terminus domain, which mediates the interaction with its specific receptors [39,40]. Adiponectin is synthetized as a 30 kDa full-length monomer (fAd), detected only in the adipocytes cytoplasm, that assembles into different oligomeric complexes before secretion [22,41]. The basic form of adiponectin complexes is a trimer (low molecular weight, LMW), which in turn can oligomerize, through disulphide bonds formation, into hexamers (middle molecular weight, MMW) and multimers (high molecular weight, HMW), consisting of 12–18 monomers [42,43,44,45]. In human plasma, adiponectin exists also as a proteolytic cleavage fragment (globular adiponectin, gAd), produced by leukocyte elastase activity, corresponding to the globular domain of the full-length protein [46,47]. Particularly, globular adiponectin has been found in serum only as a trimer, with increased potency compared to other isoforms [48,49]. Circulating adiponectin levels are regulated by different physiological, environmental, and pharmacological factors such as hormonal production, inflammatory processes, genetic polymorphisms, nutritional status, and drug administration [21]. Typically, in women adiponectin levels are significantly higher than in men, with peaks of secretion in the morning and reduced production during the night [50,51]. Some reports have widely demonstrated that the different estrogens/androgens production may influence adiponectin expression [50,51]. Particularly, in vitro and in vivo evidences showed that testosterone decreased adiponectin secretion [27,50]. Plasma adiponectin levels are also closely correlated to the circulating concentrations of other hormones. Interestingly, it has been reported as having an inverse association with adiponectin levels and fasting plasma insulin [25]. Furthermore, growth hormone (GH), glucocorticoids, prolactin down-regulated adiponectin gene expression [52]. In addition, different studies addressed pro-inflammatory cytokines released from adipose tissue, such as tumor necrosis factor α (TNFα) and IL-6, as inhibitors of adiponectin synthesis [20]. Pharmacological therapies with anti-diabetic drugs, including peroxisome proliferator-activated receptor gamma (PPAR-γ) agonists belonging to the thiazolidinedione’s class and metformin, can also modulate serum adiponectin concentrations, enhancing its expression and secretion [53,54,55,56,57]. Moreover, meta-analysis of association-studies correlated different single nucleotide polymorphisms (SNPs) in the gene encoding adiponectin, *ADIPOQ*, with reduced levels of this adipokine [58,59]. Many evidences suggested that the several active circulating forms of adiponectin exert different biological functions in specific tissues [16,38,41,60,61,62]. Adiponectin biological effects are mediated by membrane receptors. To date three different receptor subtypes have been cloned: Two classical adiponectin receptors, adiponectin receptor 1 (AdipoR1), adiponectin receptor (AdipoR2), and a non-classical third receptor, T-cadherin [28,38]. Structurally, AdipoR1 and AdipoR2 consist of seven transmembrane domains, with an opposite orientation of the C-terminus and N-terminus compared to the G-protein coupled receptors [63]. Both receptor subtypes are expressed ubiquitously, even though the levels of one always prevail over the other. AdipoR1 is abundant in endothelial cells and skeletal muscle, with AdipoR2 being more expressed in hepatocytes, and both mediate different adiponectin effects [48]. Despite a very high sequence homology of about 67%, the two classical receptor subtypes exhibit a different affinity for the several adiponectin circulating isoforms [28]. Specifically, it is well recognized that AdipoR1 displays a higher affinity for the gAd and lower affinity for the full-length molecule, while the HMW (fAd) adiponectin binds mainly AdipoR2 [64]. Contrariwise to AdipoR1 and AdipoR2, T-cadherin is a cell-surface receptor lacking a transmembrane domain. T-cadherin is predominantly expressed in endothelial cells, smooth muscle cells and in cardiomyocytes and displays affinity for the MMW and HMW but not for trimeric and globular forms of adiponectin [65,66,67]. The role of this receptor in adiponectin action has not yet been fully clarified, even though its involvement in cell adhesion and calcium-mediated signaling has been demonstrated. The lack of the intracellular domain suggested that T-cadherin acts as a co-receptor, probably competing with AdipoR1 and AdipoR2 for adiponectin binding [68]. Moreover, T-cadherin has been detected in tumor-associated endothelial cells, proposing a possible role of this receptor in tumor angiogenesis. Particularly in a mouse transgenic mammary cancer model, T-cadherin has been highlighted as a crucial factor in the cross-talk between tumor cells and the stromal compartment [69]. It is well documented that adiponectin exerts a plethora of biological effects in different target tissues, including anti-atherogenic, cardioprotective, anti-inflammatory, insulin-sensitizing, and anti-neoplastic actions. Furthermore, adiponectin regulates energy homeostasis through a direct effect on lipid metabolism and hepatic glucose output, and increasing insulin sensitivity [70]. Recent studies also highlighted that adiponectin plays a pivotal role in cell proliferation, angiogenesis, and tissue remodeling [71]. 

## 3. Adiponectin-Mediated Signaling Pathways

The various physiological effects of adiponectin depend on the several circulating isoforms of this adipokine. Nevertheless, adiponectin exerts its effects mainly by employing the Liver Kinase B 1/AMP-activated protein Kinase (LKB1/AMPK) pathway, in particular in the management of insulin in the body. Adiponectin controls glucose levels governing pancreatic β-cell proliferation and augmenting fatty acid oxidation. Moreover, adiponectin, promoting APPL-1/AMPK interaction, increased glucose uptake through glucose transporter 4 (GLUT4) [62,72,73]. It has been largely documented that insulin resistance is one of the hallmark of obese patients and low adiponectin serum levels partially contribute to this pathological state [74]. AMPK inhibits crucial signaling pathways involved in cell cycle initiation, cell growth and survival such as extracellular signal-regulated kinases 1/2 (ERK1/2), phosphatidylinositol 3-kinases (PI3K)/Protein Kinase B (Akt), c-Jun N-terminal kinase (cJNK) and signal transducer and activator of transcription 3 (STAT3) [75,76,77]. The AMPK pathway is also crucial for cell growth through the regulation of Akt/mTOR/S6K signaling. Particularly, AMPK is an upstream of tuberous sclerosis complex 2 (TSC2), a potent inhibitor of mTOR signaling. The decreased phosphorylation of AMPK results in stimulation of cell proliferation [64,78]. This adipokine also directly modulates the expression of different proteins involved in cell cycle and apoptosis (i.e., up-regulation of p53 and Bax, down-regulation of c-myc, cyclin D1 and Bcl-2) [79]. Adiponectin is also considered an anti-inflammatory cytokine for its ability to suppress the phosphorylation of nuclear factor k B (NF-kB), a transcription factor involved in several processes that regulates the activity of various pro-inflammatory mediators [80]. Furthermore, adiponectin shows anti-migratory effects through an inhibition of Wnt/β-catenin signaling pathway, fundamental for cancer progression [81]. 

## 4. Adiponectin and Female Cancers

Among the different identified adipokines, adiponectin has been largely studied for its role in influencing cancer development and progression [82,83]. Particularly, several studies reported a correlation between low levels of adiponectin in obese women and an increased risk of development and progression of several female tumors, such as cervical, ovarian, endometrial, and breast cancers. All epidemiological, in vivo and in vitro studies have been reported for each tumor.

## 5. Adiponectin in Female Cancers

### 5.1. Cervical Cancer and Adiponectin

**Figure 2:**
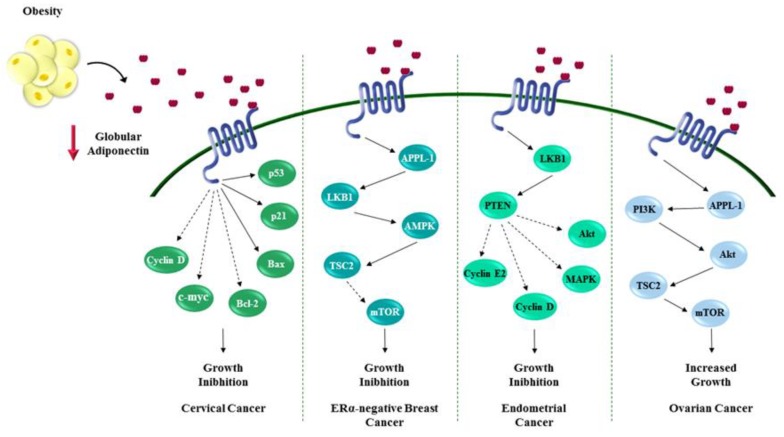
Role of adiponectin in influencing female cancers. Adiponectin, the most abundant secreted adipokine, heavily impacts the proliferation of cancer cells through several mechanisms that seem to be tumor-specific. Mainly adiponectin exerts its effects regulating cell cycle and apoptosis. The red **↓** indicates a reduction of globular adiponectin concentration. Dotted arrows show inhibition of downstream protein activation. Solid arrows mark activation of downstream proteins.

Cervical cancer is the fourth most common cancer in women both for incidence and mortality. According to WHO in 2018, 569,847 new cases are diagnosed worldwide, and 311,365 deaths [84]. The major risk factor for cervical cancer is infection with the human papillomavirus (HPV), particularly HPV16 or HPV18 [85], even though other factors may also play a role [86]. Recently obesity has been reported to increase the risk of development and progression of cervical cancer [87]. Some studies evidenced a positive association between obesity and increased risk of cervical adenocarcinoma but not squamous cell carcinoma. The increased estrogens production, due to the greater aromatase activity in adipose tissue (particularly in post-menopausal women), may explain the higher incidence of the cervical adenocarcinoma, which represents the more hormonally responsive cervical cancer type [88,89,90,91]. Although some evidences suggest that low circulating adiponectin levels related to obesity conditions may be linked to cervical malignancy, nevertheless few studies describe the molecular mechanisms though which adiponectin influences cervical cancer growth [7,92]. Noteworthy in HeLa cells, AdipoR2 mRNA expression was higher than AdipoR1, which was significantly increased in adiponectin-treated cells (10µg/mL) [93]. Xie et al. reported that low adiponectin levels inhibited the proliferation of HeLa cells, as evidenced by a significant increase in the cell population in G0/G1 phase, concomitant with a reduction of cell number in S and G2/M phases. Moreover, a down-regulation of cell cycle regulators has been reported, such as cyclin D1 and c-myc, and an activation of apoptosis, mediated by the enhanced expression of p21, p53 and Bax and the reduced level of Bcl-2 [93] (Figure 2).

### 5.2. Ovarian Cancer and Adiponectin

Ovarian cancer affects 1.6% of whole female population, according to GLOBOCAN published data in 2018 [84]. Despite a lower variability of the incidence rates compared to other gynecological malignancies, ovarian cancer remains a fatal disease, with an estimated 184,799 annual deaths [84]. Since it is asymptomatic and has not yet had specific biomarkers identified for early detection, diagnosis of most ovarian cancer cases occurs at advanced stages [84,94]. The ovary is composed of three major cell types, namely, epithelial, stromal, and germ cells, which may undergo neoplastic transformation, generating the main forms of ovarian cancer. Particularly, 80–90% of ovarian tumors originate from epithelial cells on the surface of the ovary, while stromal and germ cell cancer account only for 7 and 5% of ovarian malignancies, respectively [95]. Recently, the relationship between obesity and ovarian tumor development has become increasingly evident, particularly in post-menopausal women. Many epidemiological studies addressed obesity as an important risk factor for ovarian cancer even though the mechanisms involved in the tumorigenesis have not been fully clarified [96,97,98,99,100]. Aberrant production of hormonal factors, adipokines and cytokines, and adipose related inflammatory reactions associated with obesity may affect ovarian cancer development [101,102,103,104,105]. Interestingly, several studies linked low plasma levels of adiponectin with ovarian tumorigenesis [105,106,107]. A Kaplan–Meier survival analysis provided evidences that, in a large cohort of women affected by ovarian cancer, high leptin/adiponectin ratio correlated with a poor outcome [108]. AdipoR1 is an emerging prognostic factor for this malignancy, since many reports evidenced its down-regulation, particularly in the epithelial ovarian cancer cells [109,110]. Indeed, AdipoR1 and AdipoR2 expression is generally lower in epithelial ovarian cancer cells, such as COV434, OVCAR-3 and SKOV-3 cells, compared to granulosa tumor cells, making prognosis worse for this tumor type [109]. Abnormal activation of PI3K/Akt/mTOR cascade is well documented in ovarian cancers, and it is associated with a more aggressive phenotype [71,111]. Low adiponectin levels may favor the aberrant ovarian cancer growth, induced by the persistent activation of PI3K/Akt/mTOR signaling. Thus, it is reasonable to speculate that the increase of adiponectin levels may support the conventional ovarian cancer therapies [71,111] (Figure 2). In vitro experiments demonstrated that adiponectin inhibited growth and reversed E_2_- and IGF-1-induced cells proliferation in epithelial ovarian carcinoma. In addition, it has been demonstrated that 25 µg/mL adiponectin reduced estrogen receptor alpha (ERα), insulin growth factor 1 receptor (IGF1R), progesterone receptor (PR) mRNA, and protein expression, suggesting the functional interaction between such receptors and adiponectin signaling in epithelial ovarian cancer cells [109]. 

### 5.3. Endometrial Cancer

Endometrial cancer is the sixth most common cancer in women, with 382,069 new cases and approximately 89,929 deaths estimated worldwide in 2018 [84]. Unlike most female tumors, endometrial cancer shows a higher incidence in premenopausal women, generally nulliparous, than postmenopausal women [71]. Most of the cases are diagnosed at an early stage and surgery alone can be already effective. Nevertheless, some patients experienced disease recurrence despite adjuvant therapy [112]. Obesity is a well-recognized risk factor for endometrial carcinoma [113]. Indeed, all several bioactive molecules produced by adipose tissue, as sex steroids, insulin, insulin-like growth factors (IGFs) and the activation of their signaling sustain endometrial cancer [114,115]. Mainly, several groups investigated the correlation between adiponectin and endometrial cancer. Petridou’s group conducted the first reported case-control study in 84 women with diagnosed and histologically confirmed endometrial cancer. They suggested that in younger women (<65 years) adiponectin serum levels were inversely correlated with endometrial cancer. Moreover, they evidenced that low adiponectin concentrations correlated with high level of estrogens, insulin and IGF, molecules that sustain endometrial tumorigenesis [24]. Another study further supported these results, recognizing adiponectin as a predictive marker for endometrial cancer independently associated with obesity [116]. Also in endometrial cancer, the leptin/adiponectin ratio is recognized as a more appropriate risk marker. In fact, as described in Ashizawa’s work, higher leptin/adiponectin ratio were significantly linked with an increased probability of developing endometrial cancer. They found that the Odds Ratio (ORs) of the leptin/adiponectin ratios were higher than those of the two adipokines alone [117]. Three more recent meta-analyses have reported that adiponectin levels and leptin/adiponectin ratio are considered as predictive and prognostic biomarkers in order to guarantee early diagnosis and disease monitoring of endometrial cancer, especially in postmenopausal women [118,119,120,121]. 

Moon et al. found AdipoR1 and AdipoR2 expression in all stages of endometrial cancer as well as in non-neoplastic tissue, mainly detected in epithelial cells compared to stromal cells [122]. Moreover, both receptors have been also identified in three different established endometrial cancer cell lines, HEC-1-A, RL95-2, and KLE [122,123]. These studies reported anti-proliferative effects of adiponectin in all cell lines. At all pharmacological doses tested (ranging from 10 μg/mL to 50 μg/mL) adiponectin decreased cell growth and proliferation in a dose dependent manner (from ~20% to ~45% of reduction from low to high dose used). Cong and Moon’s groups showed that the adiponectin effects were mediated by both AdipoR1 and AdipoR2 through the activation of its canonical signaling pathway, LKB1/AMPK. Particularly, adiponectin treatment reduced ERK1/2 phosphorylation in RL95-2 cells, while it abrogated AKT phosphorylation in KLE and HEC-1-A cells depending on PTEN expression and activity. The anti-proliferative effects of adiponectin were also related to significant increase at G1/G0-phase and to a simultaneous diminution of S-phase of the treated cells [122,123]. Furthermore, adiponectin inhibited the expression of two important positive regulators of cell cycle, Cyclin D1, in KLE and HEC-1A cells, and Cyclin E2, in the RL95-2 cell line [122,123] (Figure 2). In addition, Cong et al. using an Annexin-V-FITC assay evidenced that adiponectin increased the percentage of apoptotic cells [123]. Although an important role of adiponectin in blocking endometrial cancer cell growth has been defined, and this adipokine has been suggested as a potential useful agent in the management of this neoplasia, more studies are still needed to better clarify its action.

### 5.4. Breast Cancer

**Figure 3:**
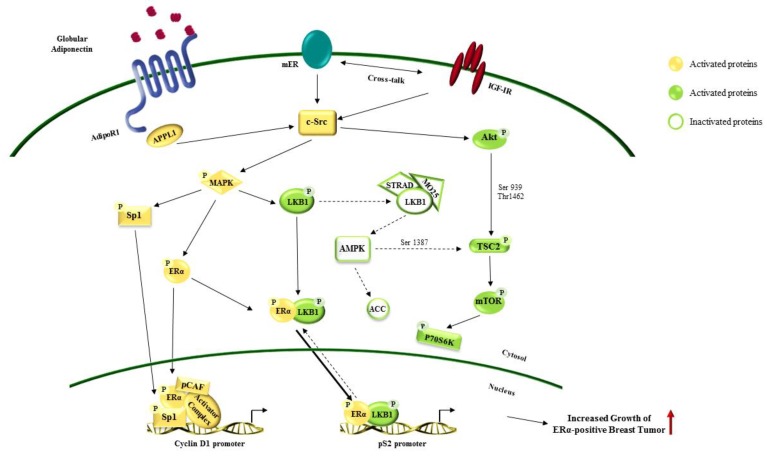
Effects of adiponectin in estrogen receptor alpha (ERα)-positive breast cancer growth. Globular adiponectin binds its receptor AdipoR1 on breast cancer cell surface. Adiponectin/AdipoR1 cross-talk with ERα and insulin growth factor 1 receptor (IGF1R) activating several downstream pathways involved in sustaining breast cancer cell growth and progression. The red **↑** Indicates increased growth of ERα-positive breast cancer. Dotted arrows show signaling inhibition. Solid arrows marks signaling activation. The arrows from cytosol to nucleus and vice versa indicate protein translocation. Solid arrows show a greater localization of the proteins in the nucleus. Dotted arrows mark a cytosolic localization.

Worldwide, breast cancer is the most common malignancy diagnosed among women (2.1 million newly cases in 2018) and it still remains one of the major causes of death for cancer in over 100 countries [84]. Several epidemiological studies reported that obesity is related with breast cancer development, progression, and poor survival [124,125,126]. Particularly it has been described that carcinoma of the breast is a complex disease in which epithelial cell-tumor microenvironment interactions play a pivotal role [127]. In this context, adipokines secreted from adipose tissue have been recognized to influence breast tumorigenesis. Among them, adiponectin is a crucial mediator in obesity-related breast cancer, since its level dramatically decreased in this pathological condition [128]. In 2003, it was reported that low serum levels of adiponectin correlates with increased breast cancer risk and contributes to a more aggressive tumor phenotype [129]. Later, several epidemiological studies and meta-analyses confirmed these findings, primarily in postmenopausal women [23,129,130,131,132]. Moreover, adiponectin levels significantly decrease with the progression of the disease while its circulating levels were not related to stage I and II of breast cancer [133]. Nevertheless, though the role of adiponectin has been well elucidated in the other female malignancies, the contribution of this adipokine in breast cancer development and progression is still controversial and under investigation. Most of the studies recognized adiponectin as a negative regulator of cancer growth in ERα-negative breast cancers; while adiponectin at relatively low concentrations might sustain tumor development and progression in ERα-positive breast cancers [81,134,135,136,137,138,139,140,141,142]. Notably, in Lam’s work it has been described that adiponectin haplodeficient tumors showed similar features to basal-like subtype tumors in terms of high proliferative activity and poor prognosis. Using MMTV–PyVT mice, they demonstrated that a decreased production of adiponectin in the tumor microenvironment contributes to induce genomic and phenotypic changes in mammary epithelial cells, in particular impacting PI3K/Akt/GSK-β-catenin signaling, a fundamental pathway that supports tumor development and progression [143]. Low serum–adiponectin levels negatively affect PTEN activation, contributing to a de-regulated PI3K/Akt/GSK-β-catenin signaling activation, confirmed in xenograft models. MDA-MB-231 cells treated with adiponectin, through a diminished Akt and GSK3-β phosphorylation, showed a reduction in breast tumorigenesis [81]. Moreover, it has been described that adiponectin interferes with Akt activation not only affecting PTEN but also through AMPK signaling. In another murine mammary tumor model it has been shown that adiponectin increased AMPK/PP2A activation that leads to dephosphorylation of Akt negatively regulating in vivo tumorgenicity [144]. Recent findings from Mauro et al. also correlate with these data; indeed, they found that in MDA-MB-231 xenograft models the pre-treatment with adiponectin (1, 5 and 30 µg/mL) reduced tumor growth at all doses tested amplifying AMPK signaling and reducing cyclin D1 expression [139,141]. Most of these results have been also described in vitro in ERα-negative breast cancer cells where adiponectin mainly serves to induce cell growth arrest and apoptosis regulating several proteins that govern cell cycle (i.e., p53, Bax, Bcl-2, c-myc and cyclin D1) [81,134,135,137,140,144,145,146] (Figure 2). Even though a large amount of authors also confirmed this pro-apoptotic role of adiponectin in ERα-positive breast cancer cells, others discovered that this adipokine might sustain tumor growth in this cell subtype. It has been reported that adiponectin fuels cell survival, migration, and differentiation of endothelial cells, and affects inflammatory cell behavior acting as a pro-angiogenic factor that contributes to breast tumor growth and progression [16,147,148]. Moreover, Mauro et al. reported that adiponectin (1 and 5 µg/mL) in MCF-7 xenografts mainly increased tumor volume concomitantly with an elevated expression of cyclin D1, high level of MAPK phosphorylation and a reduced AMPK activation [141]. Pfeleir et al. found that the combination of adiponectin and 17-β estradiol increased MCF-7 cell growth [142]. Recently, Mauro et al. in other studies fully elucidated the cross-talk between adiponectin and ERα. Firstly, they demonstrated that the multiprotein complex including AdipoR1/APPL1/c-Src/ERα/IGF-IR led to MAPK activation, in addition to adiponectin induced cyclin D1 expression at transcriptional level [140,141]. Recently they argued that this adipokine has to be considered a growth factor in ERα-positive breast cancer cells since adiponectin might impair LKB1/AMPK interaction, inducing a rapid activation of ERα and MAPK [139,140,141] (Figure 3). 

Noteworthy, it has been evidenced that adiponectin may differently modulate ERα-negative and ERα-positive breast cancer cell metabolism, an established hallmark of cancer. Cancer cells adopt “Warburg” like metabolism (i.e., anaerobic glycolysis) sustained by key regulators such as fatty acid synthase (FASN) and ACC [149,150]. LKB1/AMPK is also crucial pathway in regulating energy homeostasis, such as glucose uptake, glycolysis, fatty acid oxidation and mitochondrial biogenesis [149,151,152]. In ERα-negative breast cancer cells, adiponectin, activating AMPK/ACC, inhibits fatty acid synthesis, while in ERα-positive breast cancer cells it isn’t able to modify this process [139,141]. 

Adiponectin has been also found in the exosome, as small lipid bilayer membrane vesicles secreted by adipocytes [153], that have been recognized as important mediators of cell-to-cell communication in the complex tumor microenvironment. By transferring proteins, mRNAs, microRNAs, DNAs, lipids and transcriptional factors may induce deep changes in recipient cell’s behavior [154]. Particularly, exosomes from human adipose-derived mesenchymal stem cells (ADSCs) induce proliferation and migration in breast cancer [155] and exosomes secreted by preadipocytes also regulate breast tumor stem cell formation and migration [156]. It has been reported that adiponectin enhances exosome biogenesis and release, and although exosome cargoes adiponectin [153,157,158], more studies are still warranted to fully explain the role of this adipokine in circulating exosomes in breast cancer. 

All these evidences attempt to clarify the role of adiponectin as a mediator of breast tumorigenesis, but how adiponectin may orchestrate breast cancer is still a controversial issue that needs to be solved. 

## 6. Potential Therapeutic of Adiponectin

A balanced and healthy diet may control all the factors that have been described to sustain obesity-related disease (i.e., IGF-1, insulin, leptin) [159]. Thus, a healthy lifestyle and personal behavior should be considered as the most important prevention in this pathological condition. Indeed, the reduction of calories in diet, physical exercise and moderating consumption alcohol prevents obesity and cancer development [128]. It has been reported that vigorous aerobic exercise leads to a peak of adiponectin circulating level [160,161]. Furthermore, understanding all molecular mechanisms through which adiponectin influences tumorigenesis might provide new potential therapeutic targets. In this concern, pharmacological increase of serum adiponectin levels, up-regulation of adiponectin receptors expression, or synthesis of adiponectin receptor agonists may also be considered a promising therapeutic approach. Due to the higher frequency of breast cancer among female malignancies, most of the therapeutic strategies, aimed to ameliorate adiponectin’s response, have been proposed in breast tumor models. Interestingly, Otvos et al. developed a new adiponectin-based short peptide (H-DAsn-Ile-Pro-Nva-Leu-Tyr-DSer-Phe-Ala-DSer-NH2) named ADP 355, which could be suitable for treatment in cancer. ADP 355 showed high affinity with AdipoR1, and through the regulation of the canonical adiponectin-regulated pathways (i.e., AMPK, Akt, STAT3, and ERK1/2), reduced breast tumor growth both in in vitro and in vivo studies [162,163]. Even though, ADP 355 showed promising efficacy in several malignancies, it is particularly important to design a therapeutic strategy that also impacts leptin signaling in order to functionally and physiologically re-equilibrate the adiponectin/leptin ratio [162]. AdipoRon is an oral AdipoR1/R2 agonist that successfully reestablished adiponectin functions, mainly activating AMPK and PPAR-α pathways, in obesity-related type 2 diabetes [164]. BHD1028, BHD43, and BHD44 are three other peptides designed to fully mimic adiponectin actions. In particular, Kim et al. found that BHD1028 was the peptide that showed the highest affinity with AdipoR1 and the main activation of AMPK already at low-level concentration, more than ADP 355. In addition, the PEGylation of BHD1028 improved its stability and solubility indicating this peptide as a promising candidate for anti-diabetes and metabolic disorders [165]. PPARγ agonists, such as thiazolidinediones, rosiglitazone and pioglitazone also augment the circulating level of adiponectin through directly enhancing adiponectin gene and protein expression in a dose-dependent manner [53,166,167]. Nevertheless, the use of these drugs is still limited for their potential side effects. Another pharmacological agent that presents a tangible benefit in breast cancer treatment is the anti-diabetic drug metformin. It can prevent breast cancer cell growth through the stimulation of AMPK, inhibition of mTOR signaling, and reduction of the HER2 protein [168]. In addition, metformin reduced estrogen circulating levels via AMPK signaling, blocking aromatase promoter activity [169]. Thus, metformin appears to partially mimic adiponectin signal in the treatment of obesity-related breast cancer [56,57].

Recent findings demonstrated that adiponectin differently regulated the LKB1/AMPK/mTOR signaling in breast cancer cells. In ERα-negative cells, adiponectin phosphorylated AMPK and blocked mTOR activation, thus inhibiting breast tumor growth [139]. On the other hand, in MCF-7 cells adiponectin induced MAPK phosphorylation, which in turn transactivated ERα and activated mTOR, promoting breast tumor growth [139]. On the basis of these evidences, in a breast cancer setting it is becoming important to discriminate ERα-positive and ERα-negative tumors to specifically assess the best therapeutic approaches designed to impact adiponectin functions [64].

## 7. Conclusions

Obesity is a serious health condition and a well-recognized risk factor for many diseases, such as type 2 diabetes, cardiovascular diseases, hypertension, and cancer [6,7]. Obese female breast cancer patients are more likely to have a worse prognosis [170] and recent meta-analyses also estimated an approximately 30% increased risk of disease recurrence or death in obese versus normal weight women [171,172]. Several hypotheses have been proposed to unravel the direct link between obesity and cancer including hyperinsulinemia, estrogen signalling, inflammation and adipokine expression [173,174]. Indeed, bioactive molecules secreted from adipose tissue raised a wide spark interest in this field and among them adiponectin seems to play a potential role in influencing tumor development and progression. The effects of adiponectin, the most abundant secreted adipokine, have been largely studied in obesity-associated female-specific tumors. Although in cervical, ovarian, and endometrial cancers adiponectin exerts anti-proliferative actions, in breast cancer a new and contradictory function of this adipokine is emerging. Thus, therapeutic strategies aiming to regulate adiponectin concentrations and AdipoR1/2 activation are considered an encouraging tool in the management of obesity-related cancer, such as cervical, ovarian, endometrial, while a lot of controversial issues still remain in adiponectin treatment of breast cancer.

## Abbreviations

WHO	World Health Organization
BMI	Body Mass Index
IGF-1	Insulin-like Growth Factor-1
fAd	full-length Adiponectin
LMW	Low Molecular Weight
MMW	Middle Molecular Weight
HMW	High Molecular Weight
gAd	globular Adiponectin
TNFα	Tumor Necrosis Factor α
PPAR-γ	Peroxisome Proliferator-Activated Receptor gamma
AdipoR1	Adiponectin receptor 1
AdipoR2	Adiponectin receptor2
LKB1	Liver Kinase B 1
AMPK	AMP-activated protein Kinase
Akt	Protein Kinase B
mTOR	mammalian Target of Rapamycin
S6K	ribosomal protein S6 Kinase
TSC2	Tuberous Sclerosis Complex 2
ERK1/2	Extracellular signal-Regulated Kinases
PI3K	Phosphatidylinositol 3-Kinases
cJNK	c-Jun N-terminal kinase
STAT3	Signal Transducer and Activator of Transcription
NF-kB	Nuclear Factor k B
E2	Estradiol
ERα	Estrogen Receptor alpha
IGF1R	Insulin Growth Factor 1 Receptor
PR	Progesterone Receptor
TGF-β1	Transforming growth factor beta 1
SMAD2	Small Mother Against Decapentaplegic 2
